# Resource limitation during larval growth leads to higher flight propensity in adult beetles

**DOI:** 10.1098/rsbl.2025.0510

**Published:** 2025-10-29

**Authors:** Ori Stearns, Tomer Urca, Eran Gefen, Roi Gurka, Gal Ribak

**Affiliations:** ^1^ Department of Zoology, Tel Aviv University, Tel Aviv-Yafo, Tel Aviv District, Israel; ^2^ Department of Biology, University of Haifa-Oranim, Haifa, Israel; ^3^ School of Coastal and Marine Systems Scienceh, Coastal Carolina University, Conway, SC, USA

**Keywords:** scaling, resting metabolic rate, circadian rhythm, flight activity

## Abstract

The mango stem borer *Batocera rufomaculata* is a large beetle (Cerambycidae) exhibiting a high intra-specific variation in adult body size because of differing environmental conditions during larval growth. Previous studies revealed that smaller individuals can fly longer distances than larger ones before reaching exhaustion, a surprising fact considering that the cost of transport is expected to increase with decreased body size. We tested the flight propensity and metabolic rhythms of these beetles as a function of sex and body size. The intrinsic flight-initiating behaviour and the daily fluctuations in metabolic rate (MR) were measured over 48 h in closed arenas and in metabolic chambers, respectively. Beetles displayed a strong circadian pattern of nocturnal activity in both locomotion and MR. Smaller conspecifics were significantly more active both metabolically and behaviourally than larger ones with sex having no effect on the size-related difference. The results suggest a stronger innate drive to disperse by flight in smaller conspecifics, providing a behavioural–physiological link between environmental conditions during the larval growth period and the dispersal potential of the adults.

## 1. Introduction

Insect dispersal polymorphism is expressed by differences in dispersal-related phenotypic traits within a population, with ‘dispersers’ possessing traits better suited for long-range locomotion compared to the general population [1]. Differences can involve visible anatomical traits such as larger wings [2] and the appearance of winged morphs in a wingless population [3] or be cryptic, including behavioural or physiological changes [1,4].

Flight propensity, i.e. the tendency of an individual to voluntarily take off, may vary as a function of abiotic factors such as wind speed [5,6], temperature [7] and light intensity [8]; and biotic factors, such as intraspecific competition [9], food quality and availability [8] and reproductive state and sex [7,10–12]. Nonetheless, variation in flight propensity within a population can be seen even under fixed biotic and abiotic conditions, suggesting some innate factors within the individual may lead to variance in flight behaviour within the population [13]. The internal (genetic or phenotypic) mechanisms contributing to such intraspecific variation are unclear.

Body mass (m_b_) [14,15], egg-load [16], proportion of flight muscle to m_b_ [17], tracheal volume [18–20] and metabolic rate (MR) [21] are traits that may affect an individual’s flight performance and energetics, leading to changes in flight behaviour. These traits are subjected to allometric growth, providing a possible mechanism to generate size-related gradients in flight performance and behaviour within a population. In holometabolous insects, the development of the flight apparatus is limited to the pupa and adult stages while m_b_ growth predominantly occurs during larval stages. Therefore, size-related effects on flight can carry over from the larval stage, linking larval growth conditions with adult dispersal potential [22,23].

In the mango stem borer beetle, *Batocera rufomaculata,* females lay eggs underneath the bark of *Ficus* trees. Hatched larvae tunnel their way into the stem of the host while feeding on its tissues causing structural damage that eventually leads to the death or collapse of the host. As the larvae lack known symbionts for cellulose digestion [24,25], larvae developing inside dead trees are malnourished and emerge as smaller adults compared to larvae developing on a nutrient-rich diet in vital trees [23]. This leads to a large variation in adult m_b_ ranging from as small as 1 g up to 7 g in large individuals [14,20,23]. While all individuals are flight-capable, smaller individuals emerging from dead trees are under stronger selective pressure to disperse in search of a viable host as it is crucial source of food, mate-finding and oviposition. Prior research showed that smaller *B. rufomaculata* adults reared as larvae under poor conditions had proportionally larger flight muscles and were capable of flying greater distances in flight mills compared to larger ones [23]. Additionally, small and large individuals had similar mass-specific mechanical power output when flying at the same speed in a wind tunnel [14,26]. These empirical findings are non-intuitive because the cost of transport (the work to move 1 kg of m_b_ over 1 m) of animals tends to decrease with increasing m_b_, making locomotion proportionally costlier for smaller animals [27,28]. Indeed, free-flight MR of *B. rufomaculata* scales intraspecifically with m_b_ to the power of 0.57 [29], suggesting a higher mass-specific MR for flight in smaller individuals.

Why is it then that smaller individuals fly longer distances despite their equal or higher energetic cost? We hypothesize that the prolonged flights of smaller individuals are spurred by a higher behavioural drive for flight rather than reduced flight cost, implying a negative correlation between m_b_ and flight propensity. Using established videography methods [30,31], we measured the flight propensity of *B. rufomaculata* over the diel cycle as a function of their m_b_. A higher flight propensity in smaller individuals would suggest that differences in size dependent flight and dispersal potential are driven at least partly by behaviour. Since environmental conditions in the experiments are constant, any difference may be considered a result of carryover effects from the larval growth period. We further predict that a higher inner drive for flight would manifest itself as an increase in activity (restlessness) regardless of the flight itself. We tested this prediction by measuring the MR of the beetles over a similar period in metabolic chambers that restricted the flight of the insects. A higher MR in the flight-restricted smaller individuals would indicate an innate increase in activity tied to their restless state, presumably leading to higher flight propensity.

## 2. Material and methods

### (a) Metabolic rate assay

**Figure 1.**
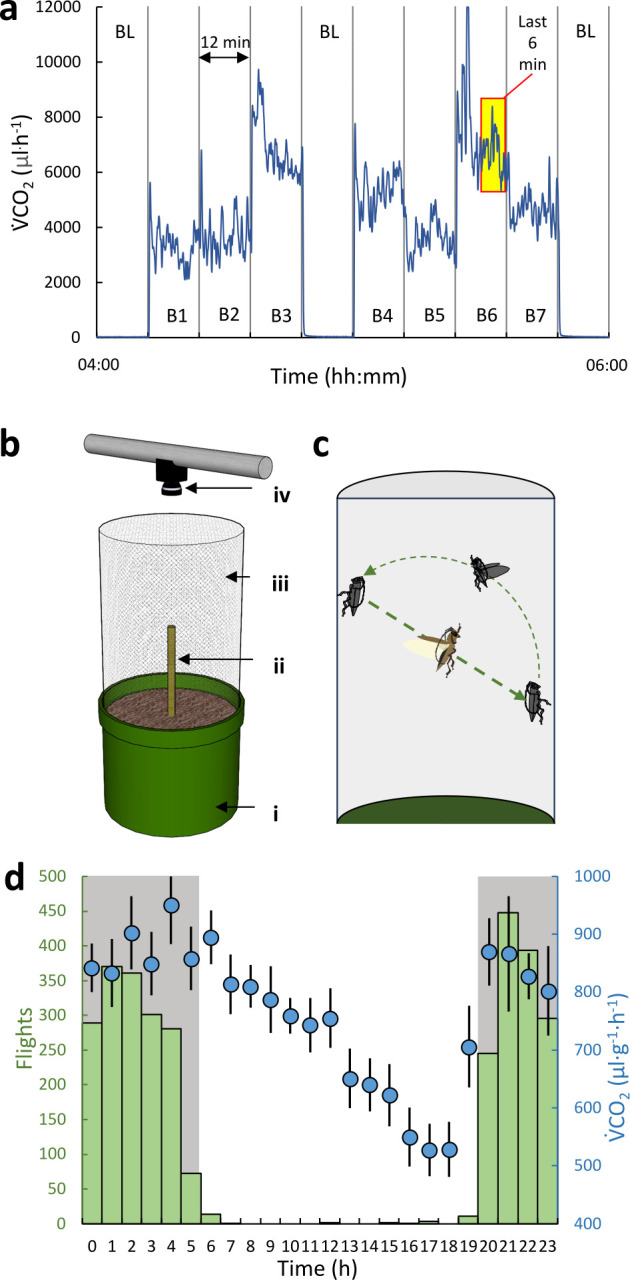
Measuring respirometry and flight activity of *B. rufomaculata*. (a) Each chamber (B1–B7) was flushed with air for 12 min, with three intermediate 12 min baseline steps (BL). For each chamber, only the last recorded 6 min were analysed (yellow box) to allow the flushing of respiratory gases remaining in the system from previous measurements. (b) The arena used for flight activity measurement comprised a soil-filled plastic pot (i), a fig tree branch (ii) metal wire mesh (iii) and a camera (iv) with IR light for night vision. (c) Flight activity was determined by counting individual flight bouts within the arena. (d) Data of both experimental set-ups were binned to 1-h periods and used to determine the daily fluctuations in mean mass-specific metabolic rate ($\dot{V}CO_{2}^{*}$, blue circles, *n* = 14) and flight activity (flight initiation counts h^−1^, green bars, *n* = 19). Whiskers denote standard deviation. Light : dark periods are indicated by the shaded (dark) and white (light) backgrounds.

Seven males and seven females adult *B. rufomaculata* were collected in August 2020 from fig trees at ‘Ayalon-Canada Park’ (N31.839208, E34.998058) in central Israel. The beetles were individually housed in boxes lined with damp paper and transported on the same day to a laboratory temperature-controlled chamber (MIR554, Panasonic, Japan) set to 25°C (± 0.2°C) with a 14 : 10 light-dark photoperiod. The beetles were fed fig branch cuttings and allowed to acclimate to lab conditions for 2 days before measurements commenced. The food was removed 8 h prior to measurements to avoid the increased MR associated with food digestion and absorption. Then, CO₂ emission rates ($\dot{V}{CO}_{2}$) were measured (as a proxy for MR) using flow-through respirometry under the same conditions. Each beetle was placed in a 40-ml custom-built metabolic chamber made from 60-ml plastic syringes. The chamber was connected to an eight-channel flow multiplexer (RM−8, Sable Systems International, USA) enabling to simultaneously measure seven beetles, with the eighth channel used for baselining. Incoming air to all chambers was scrubbed of CO₂ (Ascarite) and water vapour (silica gel) before passing through the multiplexer at 100 ml min^−1^ (MC−500SCCM-D mass flow controller; Alicat Scientific, USA), and excurrent gas from the measured chamber was passed through a CO₂ analyser (LI−7000, LI-COR, USA). CO_2_ emission rates (µl h^−1^) were calculated by multiplying the measured CO2 concentrations (ppm) by 6, based on the total gas flow rate (6 000 000 µl h^−1^). Measurements were rotated between chambers every 12 min, with three intermediate 12 min baselining steps (figure 1a), yielding a measurement from each chamber every 120 min. The analogue output from the analyzer was digitized (UI−2, SSI), recorded and analysed using Expedata software (SSI).

### (b) Behavioural assay

Infected fig trees were cut down during the winter of 2024 and stored outdoors in metal net cages. Beetles emerging from these trees were collected daily and individually housed in 1-l glass jars with fresh fig twigs for food.

Two to three weeks after emerging, the beetles (*n* = 19; 11 males, eight females) were introduced (one at a time) into cylindrical arenas (diameter 54 cm and height 39 cm) constructed out of plastic pots and metal nets (figure 1b). The pot forming the bottom of the arena was filled with soil and a freshly cut fig branch (length approx. 30 cm, diameter 1 cm) protruded from its middle acting as a perch and food source. The arenas were placed inside an outdoor glass greenhouse (2.4 × 3.6 m, height 2.2 m) located at the Garden for Zoological Research at Tel Aviv University. The glass wall and ceiling allowed for natural daylight fluctuations, which at the period of the experiment (July 2024) resulted in dawn at 05.41−06.03, and dusk at 19.34−19.52. The temperature range inside the air-conditioned greenhouse fluctuated between 24 and 36°C, resembling the air temperature outside (24–33°C) measured by a meteorological station within the garden. The greenhouse was partitioned into three separate sections, allowing for concurrent measurements of activity in three arenas while minimizing potential for interaction between the three tested insects. Nevertheless, we allocated representatives of each sex and size groups in every trio to unify any effect of social interaction, if present.

An infrared camera (RLK8−820D4-A, Reolink) above each arena recorded beetle activity continuously at four fps throughout a 48 h period. Prior to recording, we allowed each beetle 30 min to acclimate to the arena and greenhouse. In the videos, each flight initiation event (figure 1c) was manually marked with a timestamp.

### (c) Data analysis

$\dot{V}{CO}_{2}$ was measured continuously for 72 h. The data from the first and last 12 h were discarded to avoid chamber acclimation and/or desiccation and hunger effects. $\dot{V}{CO}_{2}$ was calculated after discarding data recorded during the first 6 min (figure 1) to ensure complete washout of common downstream tubing and the analyser’s optical bench cell. We calculated the beetles’ diel fluctuation of MR as $\overset{˙}{V}CO_{2\;max}-\overset{˙}{V}CO_{2\;min}$ where subscripts *max* and *min* indicate the maximal and minimal daily values of the individual’s hourly $\dot{V}{CO}_{2}$. Dividing the difference by $\overset{˙}{V}CO_{2\;min}$ of the same individuals converted this value to a dimensionless index of heightened metabolic activity. $\dot{V}{CO}_{2}$ and diel fluctuation data were log-transformed and analysed using a GLM with m_b_ as continuous predictor and sex as categorial factor.

Flight initiation events were summed over 1-h bins (figure 1d) for each beetle giving the hourly flight activity as number of flight events per hour. To test for size-related difference in flight propensity, we divided the beetles according to their m_b_ into three groups: ‘small’ (m_b_ < 4 g, *n* = 6, 4♂, 2♀, mean m_b_ = 3.12 ± 0.46 g), 'medium' (4 ≤ m_b_ < 5 g, *n* = 6, 3♂, 3♀, mean m_b_ = 4.23 ± 0.15 g) and 'large' (m_b_ > 5 g *n* = 7, 4♂, 3♀, mean m_b_ = 5.51 ± 0.37 g) and compared the total number of flights per beetle (flight activity) between groups. Due to violation of the normality assumption (Shapiro–Wilk test), we used non-parametric hypothesis testing (Kruskal–Wallis ANOVA). Effect size is reported as ${ƞ}^{2}$. Throughout the manuscript, data are presented as mean ± s.d.

## 3. Results

### (a) Metabolic rate

The beetle m_b_ ranged from 3.2 to 6.0 g with an average of 4.7 ± 1.20 and 4.8 ± 0.82 g for males (*n* = 7) and females (*n* = 7), respectively.

**Figure 2.**
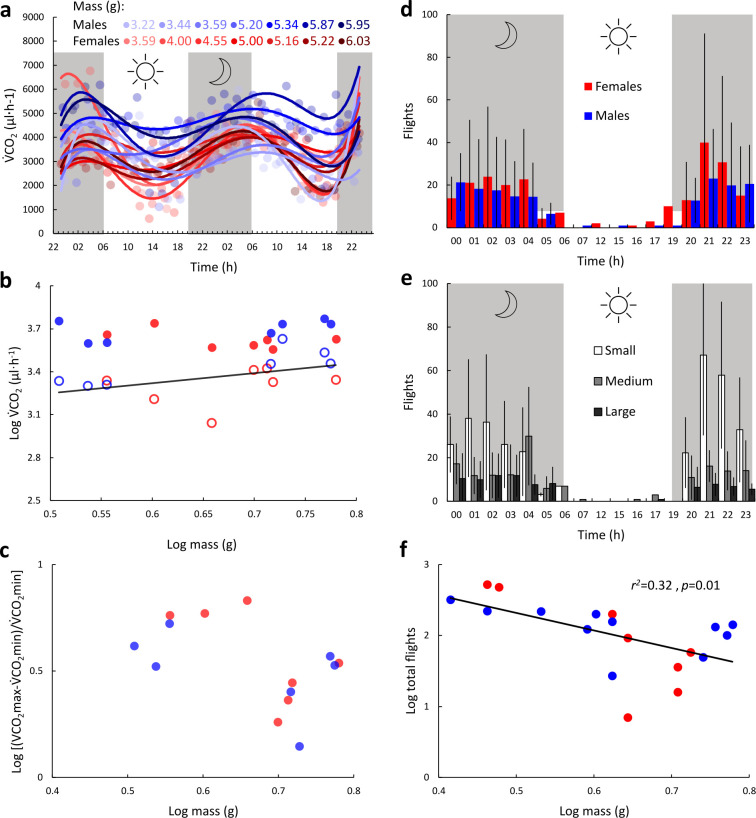
Daily metabolic and flight activity of *B. rufomaculata* as a function of sex and body mass. (a) $\dot{V}CO_{2}$ of 14 individuals throughout a 48 h period. White and shaded backgrounds correspond to light : dark periods, respectively. Circles denote the $\dot{V}CO_{2}$ of males (blue) and females (red) with colour tone indicating m_b_ (upper legend). (b) The daily maximum (filled circles) and minimum (open circles) $\dot{V}CO_{2}$ plotted against m_b_ of males (blue) and females (red). The regression line refers to the minimum values. (c) The scope (max–min) of daily CO_2_ emissions of males (blue) and females (red) as a function of their m_b_. (d) Hourly flight count of male and female beetles. Bars correspond to averages, and error bars are the standard deviation (e) The hourly average number of flights of small (white bars), medium (grey) and large (dark bars) beetles with the standard deviation denoted by the whiskers. (f) Total number of flights as a function of mass of males (blue) and females (red).

The hourly mass-specific $\dot{V}{CO}_{2}$ ($\dot{V}CO_{2}^{*}$) was the lowest between 14.00 and 18.00, subsequently increasing and reaching a maximum between 02.00 and 06.00 before decreasing back to minimal levels (figure 1d). Plotting the hourly $\dot{V}{CO}_{2}$ of all the beetles over the 48 h reveals a consistent diel pattern (figure 2a) overlaid with variance in $\dot{V}{CO}_{2}$ levels between individuals. Daily $\overset{˙}{V}CO_{2\;min}$ increased with increasing m_b_ (GLM with m_b_ as covariate, *p* = 0.03, ${ƞ}^{2}$ = 0.07, figure 2b) and was significantly lower in females compared to equally sized males (GLM with m_b_ as covariate, *p* = 0.03, ${ƞ}^{2}$=0.08) with no sex × m_b_ interaction (GLM, *p* = 0.65, ${ƞ}^{2}$ = 0.003). Daily $\overset{˙}{V}CO_{2\;max}$ did not correlate with m_b_ (GLM, m_b_ as covariate, *p* = 0.65, ${ƞ}^{2}$ = 0.001) and did not vary with sex (GLM, *p* = 0.07, ${ƞ}^{2}$ = 0.02, figure 2b). The daily fluctuation expressed as multiples of $\overset{˙}{V}CO_{2\;min}$ did not correlate with m_b_ or sex (GLM with m_b_ as covariate, *p* = 0.06 and *p* = 0.39, ${ƞ}^{2}$ = 0.14 and ${ƞ}^{2}$ = 0.03, respectively) nor displayed a significant linear decrease with m_b_ (r = 0.51, *p* = 0.06), but indicated heightened activity in beetles with m_b_ < 4.5 g (figure 2c). On average, the mass-specific $\overset{˙}{V}CO_{2\;min}$ and $\overset{˙}{V}CO_{2\;max}$ of all beetles were 471 ± 925 and 1053 ± 307 µl g^−1^ h^−1^, respectively. The mass-specific $\overset{˙}{V}CO_{2\;max}$ was 3.33 ± 1.48- and 2.05 ± 0.78-fold higher than the mass-specific $\overset{˙}{V}CO_{2\;min}$ of the same beetle for small (m_b_ < 4 g) and large (mb > 5 g) beetles, respectively. This relative increase in mass-specific $\dot{V}CO_{2}$ was significantly larger in smaller beetles (*t*‐test, *p* = 0.008, *Cohen’s d* = 0.71).

### (b) Flight activity

*B. rufomaculata* flew more at night (paired *t*‐test, *p* < 0.001, *n* = 19) with flight bouts starting around approximately 20.20 (on average 39 ± 32 min after sunset) and halting by 05.05 (48 ± 45 min before sunrise) with occasional flights occurring during daytime (figure 1d). The total number of flight initiation instances throughout the 48 h did not differ between males and females (Mann–Whitney, *p* = 0.68, figure 2d). However, the size groups differed significantly (Kruskal–Wallis, *p* = 0.01, ${{ƞ}^{2}}_{kw}=0.45$) with individuals from the ‘small’ group initiating a significantly higher number of flights than beetles from the ‘medium’ and ‘large’ groups (Wilcoxon pairwise comparisons *p* < 0.021, ‘small’: 313 ± 158, ‘medium’: 113 ± 85, ‘large’: 76 ± 48 flights, figure 2e). The total number of flights per beetle exhibited a decreasing logarithmic relationship with m_b_ (r^2^ = 0.32, *p* = 0.01, figure 2f).

## 4. Discussion

Many coleopteran species are nocturnal [32]. The respirometry and behavioural essays described here reveal that *B. rufomaculata* is no exception, with flights occurring almost exclusively at nighttime. Insect activity may increase with temperature; however, our MR measurements were performed under a fixed temperature. Thus, the metabolic fluctuations observed here likely correspond to a circadian rhythm linked to light conditions. While temperature did fluctuate in the greenhouse behavioural experiments, beetles were most active at night (when temperatures were lower). Furthermore, the correlation between diel patterns of flight activity and MR in the two experiments suggest that circadian rhythm rather than temperature (at least in the range reported here) determines the activity and flight of *B. rufomaculata* in the summer nights. In the metabolic chambers, activity increased before the dark phase (20.00 to 06.00) and decreased before the light phase (06.00 to 20.00) indicating an internal-clock-mediated anticipatory response to environmental changes as reported in flies [33] and beetle [34] species.

The threefold higher flight propensity exhibited by smaller conspecifics of *B. rufomaculata* supports our hypothesis that individuals emerging from decaying host trees possess an innate ‘dispersal restlessness’ that drives them to fly in search of a new, more viable, host. The similarity between male and female flight propensity demonstrates that size rather than sex is the major determinant of variation in flight activity. The death of the host tree should exert similar pressure for dispersal to a new host regardless of sex. In the butterfly *Lymantria dispar,* both flight propensity and distance flown in flight-mills increased with wing length [15], suggesting a positive correlation between flight propensity and flight distance. Therefore, in *B. rufomaculata* size-specific behavioural restlessness, rather than energetic efficiency, may underpin the longer cumulative flight distances observed in smaller individuals during flight-mill experiments [23]. Our MR measurements support this notion by confirming that the increased flight propensity coincides with increased MR in flight-restricted beetles, suggesting an inner drive for elevated activity.

If the lowest $\dot{V}CO_{2}$ values recorded over 48 h ($\overset{˙}{V}CO_{2\;min}$) represent times of inactivity, then $\overset{˙}{V}CO_{2\;min}$ should be close to the resting MR of the individual. The larger values of hourly $\dot{V}CO_{2}$ likely represent increased activity and $(\overset{˙}{V}CO_{2\;max}-\;\overset{˙}{V}CO_{2\;min})/\overset{˙}{V}CO_{2\;min}$ correspond to relative activity levels. Under these assumptions, smaller beetles were more active in the metabolic chambers than larger ones. We did not monitor the movement of the beetles within the metabolic chambers. Therefore, we cannot corroborate this speculation directly, but we find some support to it in the heightened locomotor activity of smaller conspecifics observed in the behavioural assay. Similarly, we cannot determine if the lower $\overset{˙}{V}CO_{2\;min}$ measured in females represents an intersex difference in standard MR (e.g. females could potentially have higher non-metabolizing mass in their stored eggs) or activity levels. The $\dot{V}CO_{2}$ measurements were performed in small cylindrical metabolic chambers of constant diameter and length required for keeping a consistent air flux through over the beetle within. The constant chamber volume left more space for smaller individuals to move potentially contributed to their higher metabolic activity. However, this is less likely since the chamber’s narrow diameter restricted the beetles’ movement to small forward and backward movements. Additionally, respiration of each beetle was measured every 2 h causing some measurements to fall between peak minimal and maximal activity levels leading to an underestimated relative increase in activity level. Nevertheless, the mass-specific $\overset{˙}{V}CO_{2\;min}$ observed here (471 ± 925 µl g^−1^ h^−1^) matches previous reports for *B. rufomaculata* measured during daytime [20], while the observed mass-specific $\overset{˙}{V}CO_{2\;max}$ (1053 ± 307 µl g^−1^ h^−1^) matches values previously measured under stress, following an injection of isotopic bicarbonate [29]. Thus, the late-night increase in MR reported here is likely associated with heightened actively while trying to exit the confinement of the metabolic chamber. Nonetheless, this heightened MR remains extremely low compared to the approximately 80-fold increase between resting and flight MR [29].

We propose that the increased flight propensity and daily fluctuations in MR of smaller individuals observed here represents a form of ‘cryptic dispersal polymorphism’ where size-related gradients in flight propensity lead to functional differences in dispersal potential between small and large beetles. All *B. rufomaculata* adults can fly, but smaller ones are more likely to disperse larger distances. Similarly, smaller conspecifics are the dispersers in the butterfly *Speyeria mormonia* [35], while larger conspecifics are the dispersers in the moth *L. dispar* [15], and medium-sized adults are the dispersers in the beetle *Librodor japonicus* [36].

In *B. rufomaculata*, adult body size is determined by conditions during larval growth [23], and our results clearly show body size-related effects on adult flight propensity and MR. Our previous work on these beetles established a link between poor larval diet and reduced adult size as well as a tendency of these smaller adults to fly longer distances [14,20,23,29]. Nevertheless, in the current work focusing on field-collected adult beetles, we cannot completely rule out the possibility of additional life-history factors that may be at play in determining flight propensity and MR. Consequently, the size-related differences in flight behaviour can be considered as a form of ‘predictive adaptive response’ [37] in which the flight of adult insects is affected by factors carried over from conditions during their larval stage rather than current conditions. Brown *et al*. [23] has previously proposed this idea to explain the proportionally larger flight-muscle mass of smaller individuals. Here, the adaptive response is at least partially behavioural. A similar phenomenon of carried over elevated flight propensity was observed in adults of the grain borer beetle, *Prostephanus truncatus* reared as larvae in higher densities [38].

Regulation of dispersal according to environmental factors is widespread in animals, with food availability being a primary driving force. In holometabolous insects, resource partitioning at the pupa stage can explain how diet and food availability lead to shifting allometries of body parts in adults [39], such as proportionally larger wings [35,40,41]. Mechanisms leading to altered behaviour across the pupae stage are less clear. One possibility is through the juvenile hormone (JH) pathway that occurs in both larvae and adults. JH has been suggested to play a critical role in the evolution of dispersal polymorphism [42,43]. In larvae, low JH levels and a deteriorating host are both known to cause premature metamorphosis, resulting in smaller adults [44,45]. In adults, lower JH levels have been shown to induce migration behaviour [43,46]. JH level also affects the MR in adult bees [47]. Such elevated flight capabilities and reduced size may come at the expense of fecundity and *vice versa* [42,48,49] as seen in phenomena such as the oogenesis flight syndrome [50]

The physiological mechanism linking conditions at larval growth with flight propensity of adults is yet to be determined. Our study underlines the importance of behaviour and innate processes in adjusting and determining the dispersal of insects. While JH analogues are in use today as pest control, they act by disrupting larval development rather than affecting adult flight behaviour. JH hormonal pathways that may carryover the effects of larval growth conditions to the dispersal drive of adults are an exciting area for future research, perhaps with potential applications in pest control through dispersal management.

## Data Availability

All data are available in the supplementary material files. Supplementary material is available online [51].
